# Prevalence and factors associated with loneliness among Indonesian female adolescents: a cross-sectional study

**DOI:** 10.1186/s12905-022-01909-5

**Published:** 2022-08-02

**Authors:** Marthoenis, Dahlia, Jacqueline Nassimbwa

**Affiliations:** 1 Universitas Syiah Kuala, Banda Aceh, Indonesia; 2 Newlife Adolescent and Youth Organization, Kampala, Uganda

**Keywords:** Loneliness, Risk behavior, Psychosocial distress, Female adolescent

## Abstract

**Background:**

Loneliness, or the perception of social disconnectedness, is a public health concern and is reported to be a frequent experience during adolescence (10–19 years). This study investigates the prevalence of loneliness and associated health-risk behaviors among Indonesian female adolescents.

**Methods:**

This was a cross-sectional study, data for which were extracted from the WHO’s Global School-based Student Health Survey (GSHS), which uses a globally standardized cross-sectional methodology to provide accurate data on behaviors and protective factors among students. Data from 4993 female students aged 13–17 years old from 74 schools were extracted from the 2015 survey in Indonesia and analyzed. Loneliness was used as a single measure, as happens in other studies using GSHS data. Descriptive analysis was done for age, socioemotional issues, health-risk behaviors, and contextual factors. This was followed by multiple logistic regression analysis to identify loneliness-associated factors.

**Results:**

Approximately 6.5% of Indonesian female adolescents experience loneliness most of the time or always. Adolescents experiencing loneliness had a higher rate of sleep disturbance (37.6%), suicide ideation (21%), suicide plan (20.8%), smoking behavior (15%), and alcohol use (15.7%) than the general population. Multiple regression analysis confirms that adolescents who have no close friends, have been bullied during the past month, experience sleep disturbance, have had suicide ideation and suicide plans, are always feeling hungry, are exposed to passive smoking, and are engaged in a sedentary lifestyle are at a higher likelihood of suffering from loneliness (*p* < 0.05). On the other hand, having kind and helpful classmates served as a useful protection against loneliness (*p* < 0.05).

**Conclusions:**

The rate of loneliness in Indonesian female adolescents is relatively lower than previously reported elsewhere. Several health-risk behaviors and contextual factors are associated with loneliness. It is important to gain insight into the exact interplay between factors and loneliness since that could inform future intervention development and lead to better well-being among female adolescents.

## Background

Loneliness in adolescents is a critical public health concern. Hawkley and Cacioppo [1] show that 80% of those under 18 years and 40% of those aged over 65 years experience loneliness globally. In Asia, 7.8% are mostly or always lonely [2]. This experience among adolescents, defined by the World Health Organization (WHO) as the phase of life between childhood and adulthood, aged 10–19 years old [3], has recently gained global recognition as a public health concern because the developmental process increases their vulnerability and frequent experience of loneliness. However, appropriate intervention guidance for this age group is lacking [4].

According to the literature, approximately one in five or six student-adolescents in Latin America, the Caribbean [5], and African countries [6, 7], experienced loneliness during the past 12 months. A lower rate (7.8%) was found in Association of Southeast Asian Nations (ASEAN) countries [8]. Additionally, loneliness affects adolescents with high occupational social class parents [9], who are of female gender, and who practice elevated smartphone and internet use [10]. The findings of a higher rate of loneliness in female adolescents have been mostly consistent among studies using the Global School-based Student Health Survey (GSHS) data [6, 8, 11]. There are some studies that found no difference between genders [7, 12], and some which show that men are disproportionately affected [13]. A recent meta-analysis focusing on gender differences in loneliness across the lifespan also found that no gender differences in loneliness changed from childhood to adolescence [14]. The use of different tools and definitions in the studies and the limited number of studies in many countries might explain any inconsistency.

While it has become a frequently raised concern among young people, loneliness is not a new phenomenon. Earlier work by Peplau and Perlman [15] summarized loneliness as an aggregate of three main characteristics: a subjective experience, an unpleasant and distressing feeling, and a consequence of personal relationship deficiency. A recent systematic review defined loneliness as “a social pain resulting from a perceived deficit in the quality or quantity of an individual’s social connection” [16]. These historical and contemporary definitions illuminate the unpleasant and distressing experiences, as well as the quality and quantity of the personal social connection. Furthermore, adolescents often describe loneliness as emptiness, boredom, isolation, and the inability to adapt to their social environment or form interpersonal relationships. They often feel lonely when rejected, isolated, and unable to have a role in their environment [17]. This shows that loneliness could be linked with their varied life events.

Various factors are associated with loneliness. At the individual level, factors such as old age, female gender [8], insomnia [18], suicidal thoughts, smoking, drinking alcohol [19], and feelings of depression and anxiety [20] have been reported. Interpersonal factors include weak peer relationships (victimization, few close friends, poor-quality friendships), poor parental relations (inconsistent parenting style, lack of parental warmth and intimacy, conflicts with parents) [8], experience of bullying [19], and higher maternal education are also reported to increase the likelihood of loneliness [18]. Considering that loneliness may increase the likelihood of unexpected mental and emotional behavior among female adolescents, understanding these factors and reducing those that are modifiable may help lower the risk. This is the first study among Indonesian female adolescents to investigate the prevalence of loneliness and associated health-risk behaviors. Our research question is, “What is the prevalence of loneliness and its associated health-risk behaviors among Indonesian adolescent females aged 13–17 years?”

## Methods

This was a cross-sectional quantitative study. The data were extracted from the most recent Indonesian Global School-based Student Health Survey (GSHS) of 2015. The GSHS uses a globally standardized cross-sectional methodology to provide accurate data on behaviors and protective factors among students [21]. The GSHS applied a two-stage cluster sampling to ensure an even representation of all students in junior and senior high schools aged 13–17 years in Indonesia. Overall, 74 junior and senior high schools participated in the study.

According to guidance from Snape and Martin [22], loneliness can be used as a single-item measure if survey space and time are major constraints. The GSHS methodology is conducted in a classroom (space constraint) and in a regular class period (time constraint) [21]. Other studies using the GSHS data have used loneliness among adolescents as a single-item measure [8, 11, 23]. In tandem with Snape and Martin [22], the GSHS assessed the presence of loneliness using one question, “In the past 12 months, how often have you felt lonely?” The answer options were “Never-1; Hardly ever-2; Occasionally-3; Some of the time-4; and Always-5”. To estimate the prevalence rate of loneliness, the response of "some of the time and/or always" is considered lonely. The age, psychosocial distress, health-risk behavior, and protective variables asked included age, having close friends, school absence, experience with physical fights, bullying, suicidal behaviors, eating behavior, tobacco and substance use, and supportive peer and family relationships. The study's detailed method and questions can be found on the WHO GSHS website [21]. The questions, response options, and coding scheme can be found in our previous report using a similar database [24].

The response rate was 94% of the total GSHS population. After excluding missing cases, 4.993 female respondents were included for statistical analysis in our study. Data were analyzed using Stata software [25]. Descriptive analysis was performed for age, socioemotional issues, health-risk behaviors, and contextual factors. The association between the dependent and independent variables was tested using the Chi-square test. Multiple regression analysis was performed to examine the association between the independent variable and loneliness. The GSHS study in Indonesia adhered to the GSHS study protocols and was approved by the government of Indonesia. The GSHS methodology can be found on the WHO website [26]. The Indonesian National Institute of Health Research and Development ethical review board approved the GSHS, and a study permit was obtained from the Indonesian Ministry of Internal Affairs.

## Results

The overall rate of loneliness among Indonesian female adolescents was 6.5%. A significantly higher rate of loneliness was found among those who used a psychostimulant drugs (e.g., amphetamine) (50%), had sleep disturbance (37.5%), had trouble from using alcohol (26.1%), had ever drunk alcohol (24.3%), had suicide ideation (21%) or a suicide plan (20.8%), have no close friends (18.6%), have attempted suicide (17.7%), always felt hungry (17.2%), currently used alcohol (15.7%), never or rarely washed hands before eating (15.3%), currently smoked cigarettes (15%), and had been physically attacked (9.8%). On the other hand, a significantly lower rate was found among adolescents with parents who understood their problem (5.4%) and those who perceived that other students were kind and helpful (5.1%). The detail of demographic characteristics, the rate of loneliness, the psychological distress, the health-risk behavior, and the protective factors of loneliness in the study sample are presented in Table 1.

**Table 1:** Demographic characteristics, the rate and associated factors of loneliness

Variable	Sample	Loneliness	*x*^2^	*p*
	n (%)	n (%)		
All	4993 (100)	323 (6.5)		
*Age group in year*				
≤ 14	3125 (62.6)	188 (6)	2.85	0.091
≥ 15	1867 (37.4)	135 (7.2)		
*Psychosocial distress*				
Have no close friend	97 (1.9)	18 (18.6)	23.88	0.0001
Missed class without permission	794 (15.9)	82 (10.3)	23.23	0.0001
Physically attacked	1110 (2.2)	109 (9.8)	25.5	0.0001
Were in physical fight	605 (12.1)	62 (10.3)	16.2	0.0001
Seriously injured	924 (20)	96 (10.4)	34.3	0.0001
Being bullied	815 (16.3)	106 (13)	68.8	0.0001
Sleep disturbance	186 (3.7)	70 (37.6)	310.1	0.0001
Suicide ideation	295 (5.9)	62 (21)	109.6	0.0001
Suicide plan	274 (5.5)	57 (20.8)	98.4	0.0001
Suicide attempt	130 (2.6)	23 (17.7)	27.7	0.0001
*Health risk behavior*				
Always hungry	169 (3.4)	29 (17.2)	33.04	0.0001
Does not eat fruit	423 (8.5)	40 (9.5)	6.82	0.009
Never or rarely was hand before eating	84 (1.7)	12 (15.3)	8.62	0.003
Currently smoking cigarette	40 (0.8)	6 (15)	4.8	0.028
Passive smoking	3771 (74.3)	268 (7.2)	13.53	0.0001
Current alcohol use	70 (1.4)	11 (15.7)	10.02	0.002
Ever drink from alcohol	37 (0.7)	9 (24.3)	19.6	0.0001
Trouble from using alcohol	23 (0.5)	6 (26.1)	14.6	0.0001
Ever use amphetamine	8 (0.2)	4 (50)	25.1	0.0001
Spend sitting more than 3 h per day	1352 (27.1)	127 (9.4)	26.2	0.0001
*Protective factors*				
Other students kind and helpful	2204 (44.1)	113 (5.1)	11.74	0.001
Parent understand the problem	1863 (37.3)	101 (5.4)	5.39	0.02

The regression analysis revealed several factors that are independently associated with loneliness. Female adolescents who experience sleep disturbance were seven times more likely to feel lonely (adjusted odds ratio [AOR] = 7.02, 95% CI = 4.78–10.30) than those who did not have any sleep problems, while those with no close friends were almost four times more likely to feel loneliness (AOR = 3.80, 95% CI = 2.06–7.02) compared to those who have a close friend. Adolescents who have ever had a suicide plan were twice as likely to feel loneliness (AOR = 2.35, 95% CI = 1.41–3.92) compared to those who have never had any suicide plan, while those with suicidal ideation were 1.6 times more likely to be lonely (AOR = 1.66, 95% CI = 1.01–2.74). Those who had been bullied were also 1.6 times more likely to feel lonely (AOR = 1.63, 95% CI = 1.20–2.22) compared to those who had never been bullied. Feeling hungry often for any reason was also associated with higher odds of loneliness (AOR = 2.03 (1.21–3.43). Likewise, those with a sedentary lifestyle (AOR = 1.53, 95% CI = 1.16–2) and those exposed to passive smoking (AOR = 1.46, 95% CI = 1.05–2.04) were also independently associated with loneliness. On the other hand, having kind and helpful friends was protective against loneliness (AOR = 0.65, 95% CI = 0.49–0.86). The regression analysis of loneliness and its associated factors are presented in Table 2.

**Table 2:** Regression analysis of loneliness associated factors among Indonesian adolescents

Variable	Unadjusted odds ratio (95% CI)	Adjusted odds ratio (95% CI)
*Psychosocial distress*		
Have no close friend	3.42 (2.02–5.79)***	3.80 (2.06–7.02)***
Missed class without permission	1.89 (1.45–2.46)***	1.22 (0.88–1.70)
Physically attacked	1.86 (1.46–2.37)***	1.16 (0.84–1.61)
Were in physical fight	1.80 (1.34–2.41)***	0.98 (0.66–1.46)
Seriously injured	2.12 (1.64–2.74)***	1.32 (0.97–1.79)
Being bullied	2.72 (2.13–3.48)***	1.63 (1.20–2.22)**
Sleep disturbance	10.86 (7.86–14.99)***	7.02 (4.78–10.30)***
Suicide Ideation	4.52 (3.32–6.14)***	1.66 (1.01–2.74)*
Suicide plan	4.39 (3.20–6.03)***	2.35 (1.41–3.92)***
Suicide attempt	3.26 (2.05–5.20)***	0.56 (0.28–1.12)
*Health risk behavior*		
Always hungry	3.19 (2.10–4.84)***	2.03 (1.21–3.43)**
Does not eat fruit	1.58 (1.11–2.23)***	1.31 (0.87–1.96)
Never or rarely was hand before eating	2.46 (1.32–4.58)**	1.67 (0.81–3.47)
Currently Smoking cigarette	2.58 (1.07–6.19)*	0.36 (0.09–1.41)
Passive smoking	1.73 (1.28–2.33)***	1.46 (1.05–2.04)*
Current alcohol use	2.75 (1.43–5.29)***	1.41 (0.53–3.74)
Ever drink from alcohol	4.75 (2.22–10.15)***	2.26 (0.62–8.19)
Trouble from using alcohol	5.18 (2.02–13.23)***	0.31 (0.04–2.50)
Ever use amphetamine	14.62 (3.64–58.75)***	5.76 (0.56–59.25)
Spend sitting more than 3 h per day	1.82 (1.44–2.29)***	1.53 (1.16–2.00)**
*Protective factors*		
Other students are kind and helpful	0.66 (0.52–0.84)***	0.65 (0.49–0.86)**
Parent understand the problem	0.75 (0.58–0.95)**	0.87 (0.65–1.14)

*p < 0.05, **p < 0.01, ***p < 0.001

## Discussion

This study investigates the prevalence and associated factors of loneliness among female school-going adolescents in Indonesia. A prevalence of 6.5% of feeling mostly or always lonely was found in this study population. This finding is lower than that reported among female adolescents in ASEAN countries at 7.8% [8] and is even significantly lower than in some African countries, such as Morocco (25.1%) [6] and Tanzania (17.4%) [7]. It is also significantly lower than in the Latin American and the Caribbean countries (14.6%) [5], as well as the USA (14.4%) and Russia (14.7%) [27]. While this prevalence should be a concern, the lower rate in Southeast Asia compared to other regions may be explained by social connectedness and high income.

In a recent meta-analysis, Eccles and Qualter [4] show that social cohesion is a strong factor in alleviating loneliness. Unlike Western cultures, which emphasize independence and individualism, Asian societies, including Indonesia, have evolved into a more socio-centric, collectivistic, and interdependent culture [28]. This could explain the lower prevalence of loneliness among Indonesian adolescent girls and is reflected in the even lower rates found among adolescents with supportive parents and close and kind friends. The significant contribution of positive social relationships has also been consistently reported elsewhere [5, 6, 11], and our study shows that adolescent girls without close friends are 3.8 times more likely to experience loneliness.

Our study further shows the association between loneliness and several health-risk behaviors such as substance use (psychostimulants, drug use), psychosocial distress (being bullied, suicide planning and ideation, sleep disorders, no close friends), and a sedentary lifestyle. According to Peltzer and Pengpid [2], substance abuse is a way of coping with loneliness, and loneliness is a predictor of depression and suicide. Additionally, being bullied relates to the inability to form friendships [2, 29, 30]. Shaheen et al. [30] show that Jordanian adolescents that reported having reduced support from family or friends experienced more bullying. Kendrick et al. [29], on the other hand, found that the quality of one’s friends is protective against bullying. We found that adolescent girls bullied in the past month are 1.6 times more likely to experience loneliness than those who have never been bullied. While our study did not measure the association between social connectedness and bullying, the findings of the association between social disconnectedness, bullying, and the experience of loneliness indicate that this relationship might exist in our study sample. Nevertheless, loneliness is reported to be both a cause and a consequence of either being bullied [29, 30] or suicidal behavior [31–33]. The direction of association for either variable is unclear and would warrant additional investigation.

We also found associations between loneliness, as well as social and environmental factors such as hunger and passive smoking. The experience of hunger in this study is an indicator of the food insecurity situation and a proxy for the socioeconomic status of the study population. The finding that food insecurity may cause loneliness has also been found among Tanzanian adolescents [7, 34]. These authors link lower class socioeconomic status and limited family support to the likelihood of being hungry and also highlight the negative effect hunger has on mental health. A different study, even though in an older US population, shows that loneliness increases the likelihood of being hungry and recommends strengthening the social support system for this group [35]. On the other hand, passive smoking is common, and smoking regulation in Indonesia is generally poor. The smokers, mostly males or fathers, smoke inside the home [36] and expose their lonely female adolescents who stay mostly at home. The increased odds of loneliness from passive smoking found in our study have also been consistent with previous reports, especially those using the WHO-GSHS data [6, 11]. However, the cause and effect of hunger, passive smoking and loneliness in adolescent populations is not well understood and requires further research.

The prevalence of loneliness among adolescent females may be lower in Indonesia but should still be a concern. Peltzer and Pengpid [37], in their study on loneliness in the general population of Indonesia, show that adolescents and the older population have the highest prevalence in the country. Considering the possible increased risk faced by female adolescents, national-level efforts should be age-specific and gendered.

This study's limitations include that it was focused on adolescent females in schools because the GSHS is only conducted in those environments and, as such, findings do not represent all female adolescents in the country. The inclusion of female adolescents not attending school may generate different results. Additionally, the absence of covariates or use of loneliness as a single-item measure has been previously reported to be limiting as a qualitative indicator. The cross-sectional design also makes it difficult to measure causation. Future studies on loneliness may consider collecting primary data through longitudinal studies instead of using GSHS data.

## Conclusion

This study shows that the prevalence of loneliness among female adolescents in Indonesia is lower than reported in studies from other countries. Nevertheless, it should be a concern because of the likely disproportionate risk they face. The associated factors are numerous, interrelated, and bidirectional as causes and effects of loneliness. Addressing the negative factors together may help to avert loneliness and the consequent effects in the short and long term. Behaviors that can be explored at the individual level include strengthening social cohesion, such as having close friends or supportive classmates, and adopting healthy sleeping habits. Institutions of learning should enact rules that promote nutrition and deter bullying. Authorities should closely monitor and support adolescent girls who are reported to be or appear to be lonely. Generally, we show in this study that loneliness exists among Indonesian female adolescents and that the government should sensitize the public about associated factors to manage it and prevent its escalation.

## Data Availability

The datasets used during the current study are available from the WHO GSHS website: https://www.who.int/ncds/surveillance/gshs/en/.

## Abbreviations

ASEAN: Association of Southeast Asian Nations
AOR: Adjusted odds ratio
GSHS: Global School-based Student Health Survey
WHO: World Health Organization
